# New Technologies to Decontaminate Pollutants in Water: A Report about the State of the Art

**DOI:** 10.3390/toxics10030128

**Published:** 2022-03-07

**Authors:** Fabrizio Olivito, Pravin Jagdale

**Affiliations:** 1 Laboratory of Organic Synthesis and Chemical Preparations (LabOrSy), Department of Chemistry and Chemical Technologies, University of Calabria, 87036 Rende, Italy; 2 Center for Sustainable Future Technologies (IIT@PoliTO), Italian Institute of Technology (IIT), 10144 Torino, Italy

The growing increase in the world population was accompanied by a massive development of industrialization. The production of food and all types of utility is part of a cyclical mechanism that involves the consumption of raw materials and the consequential production of waste that must be disposed of or recycled. Unfortunately, to this day the quantity of products that are not recycled because they end up in the environment is conspicuous [1,2]. The cause of the contamination of our planet is manifold and the pharmaceutical, agro-food, construction and energy industries are among the many examples [3]. Given the growing concern related to global warming and the damage that then affects the planet itself and all living beings, the words sustainable and renewable are increasingly common today [4]. The transition to an eco-sustainable future is the only way out to ensure a better world for future generations. Pollutants entered our eco-system through land, water and air. Most of these pollutants are chemical compounds in the form of solids, liquids or gases. Scientists have been developing eco-friendly strategies for the removal of pollutants for several decades [5,6]. The removal of the latter allows for the creation of a closed recycling cycle that does not negatively affect the environment. Converting abundant raw materials into substances with multiple uses is one of the most explored scientific fields of our day [7,8,9]. Various products, such as food or agricultural waste, are used as absorbents for pollutants [10,11]. Biomass is one of the most important examples, along with other natural products [12]. In addition, there are also synthetic materials whose synthetic procedures do not negatively impact the environment, such as polymers [13,14]. This special issue “New technologies to decontaminate pollutants in water” focuses on the removal of pollutants from one of the most precious things we have on our planet, water. This editorial aims to summarize the salient points of the research articles and reviews published in this special issue that represent the latest generation research in this sector. The first research article by X. Sheng et al. is focused on the effects that micro-plastics have on the absorption of other pollutants. Multi-walled carbon nanotubes were used as adsorbent in aqueous samples and carbamazepine as the contaminant. Different factors were investigated including the quantity of micro-plastics, the pH of the medium and the concentration of carbamazepine in solution. The results reveal the negative effects that micro-plastics have in inhibiting the absorption of pollutants by carbon materials [15]. R. Balint et al. investigated the synthesis of a new promising material composed of Bismuth (V) for the adsorption of arsenic from water. The adsorbent was synthesized by solid-state reaction in which Bi(V) sites play the central role in the defective structure of the material. The adsorbent was tested for the removal of arsenite and arsenate with excellent adsorption capacity. The kinetic and adsorption isotherm of the process was thoroughly investigated and discussed. The kinetic follows a presudo-second order model, while isotherm follows the Langmuir model. In addition, the adsorption was also evaluated in the presence of other ions to assess any competitive effect. In conclusion the Authors developed an innovative adsorbent for the effective removal of As(III) and As(V) with an easy regeneration and multiple reuse [16]. In another work, A. De Nino et al. focused their attention on the preparation of composite polyurethane foams for the removal of dangerous oils from water surfaces. They prepared hydrophobic materials by micro-particles surface coating using silica and activated carbon. The adsorption capacity was tested with diesel, gasoline and oil engine. The oil adsorption capacity in diesel was improved up to 36% using the composite with silica and up to 50% using the composite with activated carbon. These materials showed optimal performances also in oil/water mixtures including fresh-water and sea-water samples. The composite with activated carbon showed the best adsorption capacity. The oil phases can be recovered by a simple centrifugation and the same it is valid for the composites that can be regenerated many times [17]. Fatemeh Biglar et al. tackled the problem of removing toxic dyes from the environment using the photochemical approach. In particular the study focuses on the Reactive red 198 (RR198) removal by a falling-film photo reactor containing ZnO-Nd nano-catalysts. This material was synthesized for the first time by a combustion method differently from the conventional approaches. The characterization was discussed thoroughly using different techniques including using FESEM, XRD, Bandgap calculation, and FTIR analysis. Different important parameters were taken into account, as the amount of H_2_O_2_, the quantity of catalyst, pH, and the initial concentration of dye. This methodology represents a valid and innovative breakthrough for the decolourisation of samples contaminated by dyes [18]. E. Dell’Armi et al. reported an interesting strategy to solve the problem of water samples contaminated by halogenated hydrocarbons. The study is based on the reductive dechlorinating reaction (RD) induced by microorganisms. The reaction is allowed by the release of molecular hydrogen by fermentable organic substances. Three different electron donors constituted by lactate, hydrogen, and a bio cathode of a bio electrochemical cell have been investigated in the batch experiments. The study proved that through an innovative technology, the bio electrochemical system reaches comparable efficiencies with a fermentable substrate without the use of other chemicals [19]. In another research article Hongyou Wan et al. analysed the potential of metal-organic frameworks for the removal of polystyrene micro-plastic from the environment. They employed a new type of material called ZIF-67, reaching an adsorption up to 92.1% in 20 min at 298 K. The adsorption capacity was investigated at different pH with the best performance at pH of 8. The main driving forces of this adsorption are hydrogen bonds, stacking and electrostatic interactions. These findings represent a step forward in the field of removing micro-plastics with innovative and eco-sustainable materials [20]. In the experimental report by P. Guidi et al. they have described and studied experimentally, the damage that spills of petroleum and derived oils in water can create on the environment and in particular on living organisms. Due to the fact that some petroleum-derived hydrocarbons are classified as mutagenic and carcinogenic contaminants, mostly related to the water soluble fraction, they investigated the positive effect of pure anatase titanium nanoparticles (n-TiO_2_) to lower the DNA damage induced by the exposure of petroleum-derived hydrocarbons on Dicentrarchus labrax. This laboratory-scale study can provide important information on the protection of marine organisms and beyond [21]. Subhash Chandra et al. in a concise and meaningful review reported the recent techniques on the removal of sulfamethoxazole by photocatalytic degradation using biochar-supported TiO_2_-based nanocomposites. The problem of the massive administration of antibiotics to humans and animals and their easy bio-accumulation promotes more and more phenomena of antibiotic-resistance. Sulfamethoxazole is one of the most used antibiotics for the urinary, respiratory, and intestinal infections and as a supplement in animal and fish farms. Bio-char is an important organic materials derived by renewable sources and its composites can become in the next future, sustainable materials for various applications such as the photo-catalytic degradation of pharmaceuticals. This review collects the recent research about the synthetic techniques and applications of composites materials made of TiO_2_ nanoparticles and biochar [22]. In conclusion, we think that this editorial and all the related research works can offer a valid starting point to open new paths in the field of clean, sustainable and renewable technologies to remove pollutants.
